# Photoelectrocatalytic Dioxygen Reduction Based on a Novel Thiophene-Functionalized Tricarbonylchloro(1,10-phenanthroline)rhenium(I)

**DOI:** 10.3390/molecules28073229

**Published:** 2023-04-04

**Authors:** Yu-Qin Li, Ke-Zhi Wang

**Affiliations:** Beijing Key Laboratory of Energy Conversion and Storage Materials, College of Chemistry, Beijing Normal University, Beijing 100875, China

**Keywords:** rhenium complex, photoelectrochemical property, thiophene group, modified electrode, photoelectrochemical oxygen reduction

## Abstract

A novel Re (I) complex of [Re(CO)_3_Cl(L)], {L = 2-([2,2’-bithiophen]-5-yl)-1-phenyl-1*H*-imidazo [4,5-*f*][1,10]phenanthroline}, was synthesized, and its optical (UV–Visible absorption and emission spectroscopy), cyclovoltammetric and photoelectrochemical oxygen reduction properties were studied. The geometric and electronic properties were also investigated by density functional theory calculations. It was found that the ITO electrode coated with drop-casted [Re(CO)_3_Cl(L)] film exhibited cathodic photocurrent generation characteristics. The illuminated film exhibited a maximum cathodic photocurrent up to 30.4 μA/cm^2^ with an illumination intensity of 100 mW/cm^2^ white light at a bias potential of −0.4 V vs. SCE in O_2_-saturated electrolyte solution, which was reduced by 5.1-fold when thoroughly deoxygenated electrolyte solution was used, signaling that the electrode performed well on the photoelectrochemical oxygen reduction. The photo-electrocatalytic hydrogen peroxide production was proved with a maximum H_2_O_2_ concentration of 6.39 μM during 5 h of the photoelectrocatalytic process. This work would guide the construction of more efficient rhenium-based photo(electro)catalytic molecular systems for O_2_ sensing, hydrogen peroxide production and other types of photoelectrochemical energy conversion and storage.

## 1. Introduction

The most severe challenges facing humanity in the 21st century may be energy shortages due to our dependence on limited fossil fuel resources [1,2,3]. To address this challenge, there has been significant scientific interest in finding alternative ways to harvest, convert and store energy. Considering the abundant and renewable nature of sunlight, the direct conversion of it into energy stored in chemical bonds is believed to be a particularly promising way to overcome the problem. Recently, the photoelectrocatalytic production of H_2_O_2_ has attracted increasing interest in terms of energy and environmental contexts [4,5,6,7,8,9].

Nowadays, substantial research interest has been attracted to rhenium coordination complexes based on their excellent, promising applications in radiopharmaceuticals and diagnostics [10], bioinorganic chemistry [11], probes of environmental changes [12], electroluminescent materials [13], catalysis [14], photoelectrochemistry [15] and photo(electro) catalysis [16,17,18,19,20,21,22]. Especially in the molecular catalysts, the tricarbonyl Re complexes have been well-known to show good electrocatalytic and photocatalytic activities for the reduction of CO_2_ to CO with high efficiency, stability and product selectivity [16,17,18,19,20,21,22]. However, the photoelectrochemical H_2_O_2_ production has not yet been reported for this family of molecular materials.

It is well known that photo(electro)catalytic performance could be affected by visible-light absorption, charge separation and interface chemical reaction [7]. In addition, it was reported that an increase in π-conjugation could extend the absorption band into the visible region, which could also promote the separation of photo-generated charges, contributing to enhanced photo(electro)catalytic performance [21,22]. The thiophene group has attracted much attention for introduction into Ru(II) polypyridyl complexes [23,24] due to its enhanced light absorption, good electronic conductivity and charge transport properties [25,26,27,28,29,30], but thiophene group-grafted tricarbonyl Re complexes have not received enough attention [16,31]. Wang et al. reported a tricarbonyl Re complex with thiophene-substituted imidazole [4,5-*f*]-1,10-phenanthroline ligand, and density functional theory calculation showed that the complex should possess good hole-transfer ability [31]. Sun et al. reported three novel Re complexes with thiophene-substituted bipyridine ligands and their electropolymerized films on a glass carbon electrode. The film electrode showed relatively high stability and significant electrocatalytic reduction of CO_2_ to CO [16]. On the other hand, the stable immobilization of the Re complexes on solid supports is very significant for practical electrocatalytic and photoelectrocatalytic applications [32]. The commonly used film fabrication techniques such as covalent and electrostatic self-assemblies have been proved to be rather successful, but still face great challenges for film stability [33]. The drop-casting that only needs to pipette a compound dissolved in a solvent onto a surface and leave it to dry has been evidenced to be a simple, easy, rapid and low-cost technique for the fabrication of thin films of sparingly soluble molecules with high adhesion to substrate surfaces [15,34,35,36]. Wada et al. reported drop-casted Ru(II) complex-based films with highly efficient and durable electrocatalytic oxidation of water to dioxygen [35]. Zuo et al. reported efficient perovskite solar cells that were made by using a drop-casting technique [36]. We also previously reported Ru(II) [37] and Re(I) [15] complex-based drop-casted films with highly producible photocurrent generation and switching properties. In this paper, we would like to report a drop-casted film of a bithiophene-grafted Re(I) complex with interesting photocurrent generation and photoelectrocatalytic H_2_O_2_ properties.

## 2. Materials and Methods

### 2.1. Instrumentation

^1^H NMR spectra were acquired on Bruker DRX-400 or 600 NMR spectrometers, with Me_2_SO-d_6_ as the solvent. Chemical shifts (δ) were reported in parts per million (ppm), with tetramethylsilane as the internal standard. The infrared (IR) spectrum was measured using a Nicolet Avatar 360FT-IR spectrometer with a KBr disk. UV–Visible (UV–vis) absorption spectra were measured on a GBC Cintra 10e UV–vis spectrophotometer. Fluorescence spectra were recorded on a Varian Cary Eclipse fluorescence spectrometer.

### 2.2. Materials

2-([2,2’-Bithiophen]-5-yl)-1-phenyl-1*H*-imidazo [4,5-*f*][1,10]phenanthroline (L) was synthesized according to a modified method we published before [23]. Other reagents and solvents were used as received without further purification.

*Synthesis of [Re(CO)_3_Cl(L)]*. A toluene (6 mL) solution containing [Re(CO)_5_Cl] (118 mg, 0.326 mmol) and L (150 mg, 0.326 μmol) was refluxed at 110 °C for 6 h under a dinitrogen atmosphere. After cooling to room temperature, the precipitated solid was collected by filtration, washed with toluene, and dried in vacuo. The resulting crude product was further purified by column chromatography on silica using mixed solvents of dichloromethane-ethyl acetate (*v*/*v*, 4:1) as the eluent. The pure target complex was acquired as a yellow powder. Yield: 197 mg (79%). ^1^H NMR (600 MHz, DMSO-*d*_6_) δ 9.44 (d, *J* = 5.1 Hz, 1H), 9.36 (d, *J* = 8.1 Hz, 1H), 9.30 (d, *J* = 5.1 Hz, 1H), 8.21 (dd, *J* = 8.3, 5.1 Hz, 1H), 7.98 (d, *J* = 7.7 Hz, 1H), 7.93 (d, *J* = 7.0 Hz, 1H), 7.90 (d, *J* = 7.4 Hz, 3H), 7.84 (dd, *J* = 8.6, 5.0 Hz, 1H), 7.60 (dd, *J* = 13.4, 6.9 Hz, 2H), 7.39 (d, *J* = 3.6 Hz, 1H), 7.22 (d, *J* = 4.0 Hz, 1H), 7.12 (t, *J* = 4.4 Hz, 1H), 6.64 ppm (d, *J* = 4.1 Hz, 1H) (Figure S1,bottom). C_30_H_16_ClN_4_O_3_ReS_2_ (765.991): C 47.02, H 2.10, N 7.31; found C 47.18, H 2.03, N 7.42. ESI-MS: *m*/*z* = 766.995 [M + H^+^]^+^. IR in KBr pellet (ν/cm^−1^): 2927, 2868, 2016, 1922, 1887, 1607, 1478, 1455, 1396, 1361, 812, 731, 696 cm^−1^. 

### 2.3. Fabrication of the Modified Electrode

Indium–Tin oxide (ITO)-coated glass substrates used for the drop-casted films were treated as shown below. First, the ITO substrates were ultrasonicated in detergent for 20 min and then in de-ionized water for 5 min. Second, the thus-treated ITO substrates were soaked in a mixture of 25% NH_3_•H_2_O-30% H_2_O_2_-deionized water (*v*/*v*/*v*, 1/1/5) and boiled at 70 °C for 20 min. Finally, the ITO substrates were thoroughly rinsed with copious deionized water and ethanol and then dried in a blast oven for further use. The ITO electrodes coated with [Re(CO)_3_Cl(L)] were prepared according to the reported method [36] by drop-casting dichloromethane solutions of [Re(CO)_3_Cl(L)] (1.38 μM, if concentrations otherwise stated) onto pre-treated ITO substrates by a microsyringe and drying in an air atmosphere. 

### 2.4. Optical, Electrochemical and Photoelectrochemical (PEC) Experiments

Based on the method described by Demas et al. [38], the emission quantum yield (*Φ*_u_) of [Re(CO)_3_Cl(L)] was derived according to Equation (1) by using an air-equilibrated water solution of [Ru(bpy)_3_]^2+^ (bpy = 2,2′-bipyridine) as the standard sample (*Φ*_s_ = 0.028 [39]):

(1) $$\Phi_{u}=\frac{I_{u}}{I_{S}}\times \frac{A_{s}}{A_{u}}\times \frac{{n_{u}}^{2}}{{n_{s}}^{2}}\times \Phi_{s}$$

where the subscripts *u* and *s* refer to unknown and standard samples, respectively, *I* is integrated emission intensities, *A* (<0.1) is the solution absorbances at the excitation wavelength and *n* denotes the refractive indices of the solvents.

Cyclic voltammetry and PEC measurements were completed at room temperature using a CHI601 electrochemical analyzer. Cyclic voltammetry experiments were performed employing an ITO, an Ag wire and a Pt disk as the working electrode, the pseudo-reference electrode and the counter electrode, respectively, in a CH_2_Cl_2_ solution of [Re(CO)_3_Cl(L)] with 0.1 M Bu_4_NPF_6_ as the supporting electrolyte. The PEC measurements were performed using a [Re(CO)_3_Cl(L)] drop-casted ITO electrode as the working electrode, a saturated calomel electrode (SCE) as the reference electrode and the Pt plate electrode as the counter electrode, which were immersed in an Na_2_SO_4_ aqueous solution (0.1 M). The working electrode that was placed at a position of an approximately 15 cm distance between the film surface and a 500 W xenon lamp (Changtuo Co. Ltd., Beijing, China) was irradiated with an effective area of 0.28 cm^2^ by a white light produced from the xenon lamp light source equipped with an infrared cut-off filter (730 nm > λ > 325 nm). The light intensities were measured using an ST-900M photometer (Photoelectric Instrument Factory, Beijing Normal University). The uncertainties for the photocurrents/photocurrent densities reported were estimated to be less than 15%. The H_2_O_2_ produced in PEC cells was quantified by means of the spectrophotometric molybdenum-triiodide method [40].

### 2.5. Computational Details

The ground state geometry optimization of the [Re(CO)_3_Cl(L)] monomer and its dimer was performed with the Gaussian 09 [41], using a density functional theory (DFT)-based method with the hybrid B3LYP functional [42,43]. In addition, the LanL2DZ basis set was adopted for the complexed rhenium metal [44,45], while the 6-31G(d) basis set was applied for C, H, N, Cl and O [46,47]. The optimized structure was confirmed through vibration analysis, and the frontier orbital distribution was explained using Löwdin population analysis [48].

## 3. Results and Discussions

### 3.1. Synthesis and Characterization

**Scheme 1:**
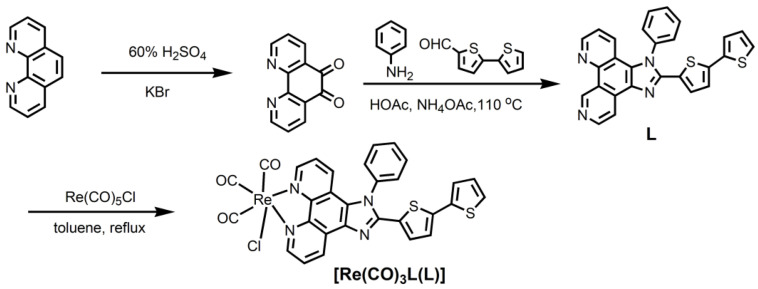
The synthetic route to [Re(CO)_3_Cl(L)].

L and its Re(I) complex of [Re(CO)_3_Cl(L)] were synthesized according to the routes shown in Scheme 1. The ligand L was synthesized according to a modified method we published before [23]. [Re(CO)_3_Cl(L)] was synthesized as previously reported [49] by reacting equimolar [Re(CO)_5_Cl] and L in toluene, and it was easily purified by a simple washing with toluene, followed by silica gel column chromatography with dichloromethane-ethyl acetate (*v/v*, 4:1) as an eluent. [Re(CO)_3_Cl(L)] was characterized by elemental analysis and ^1^H NMR (Supporting Information, Figure S1), infrared (Figure S2) and mass spectroscopies. The experimental values of the C, H, N elemental analysis match their theoretical values. The integrated areas of the proton resonance peaks in the ^1^H NMR spectra of both L and [Re(CO)_3_Cl(L)] correspond to 16 protons, which are consistent with the theoretical prediction. The chemical shifts of the protons adjacent to two coordinating nitrogen atoms of 1,10-phenanthroline in [Re(CO)_3_Cl(L)] were observed to experience more evident shifts relative to those in L. In addition, the corresponding protons at the other positions of L and [Re(CO)_3_Cl(L)] did not show significant differences in their chemical shifts. The positive ion electrospray ionization mass spectrum of the complex showed a strong molecular ion peak centered at *m*/*z* = 766.995, which is attributable to [M + H^+^]^+^, with a calculated m/z value of 766.991. The IR spectrum (Figure S2) showed three strong absorption peaks at 2016, 1922 and 1887 cm^−1^, which were attributed to the stretching vibrations of three carbonyl groups.

### 3.2. Optical Properties

**Figure 1:**
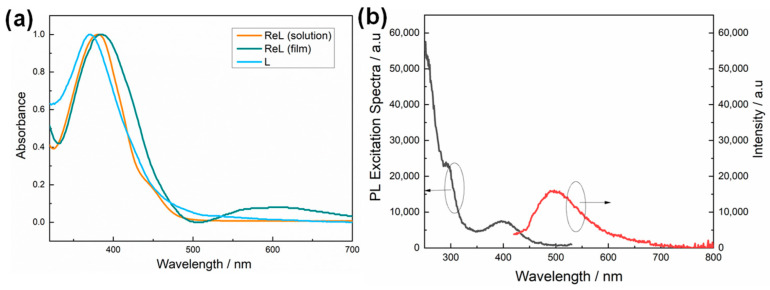
(**a**) Comparison of normalized UV–vis absorption spectra of ligand L (blue line) and [Re(CO)_3_Cl(L)] in drop-casted film (green line) and DCM solution (red line) at room temperature. (**b**) Excitation (black line, λ_em_ at 500 nm) and emission (red line, λ_ex_ at 400 nm) spectra of [Re(CO)_3_Cl(L)] in DCM solution (1.38 μM).

The UV–vis absorption and photoluminescence spectra of L and [Re(CO)_3_Cl(L)] in dichloromethane (DCM) and the drop-casted film on the indium–tin oxide (ITO)-coated glass substrate are compared in Figure 1. As shown in Figure 1a, [Re(CO)_3_Cl(L)] exhibited a metal-to-ligand charge-transfer (MLCT) absorption shoulder band [50,51] centered at ~450 nm and an intraligand π-π* absorption band centered at 380 nm, which is bathochromically shifted by 10 nm as compared to the π-π* absorption band at 370 nm observed for L [23]. In addition, the most prominent characteristic of the film is the appearance of a new absorption band centered at 600 nm, in addition to the feature that the π-π* absorption is red-shifted and significantly broadened with respect to the spectrum of [Re(CO)_3_Cl(L)] in DCM, indicating the formation of *J*-aggregates in the film [52]. Based on the onset absorption at 488 nm for [Re(CO)_3_Cl(L)] in DCM, the optical gap of the complex was roughly estimated to be 2.54 eV.

The photoluminescence excitation and emission spectra of [Re(CO)_3_Cl(L)] in DCM are shown in Figure 1b. The excitation spectrum (λ_em_ = 500 nm) showed a distinct band at 400 nm with a feature close to that of the UV–vis absorption spectrum, indicating that the photoluminescence originated from the excitation of [Re(CO)_3_Cl(L)] rather than impurity. Upon excitation at 400 nm, [Re(CO)_3_Cl(L)] in DCM displayed a broad and structureless emission band at 500 nm, which could be ascribed to the luminescence from the ^3^MLCT excited state [53,54,55].

**Figure 2:**
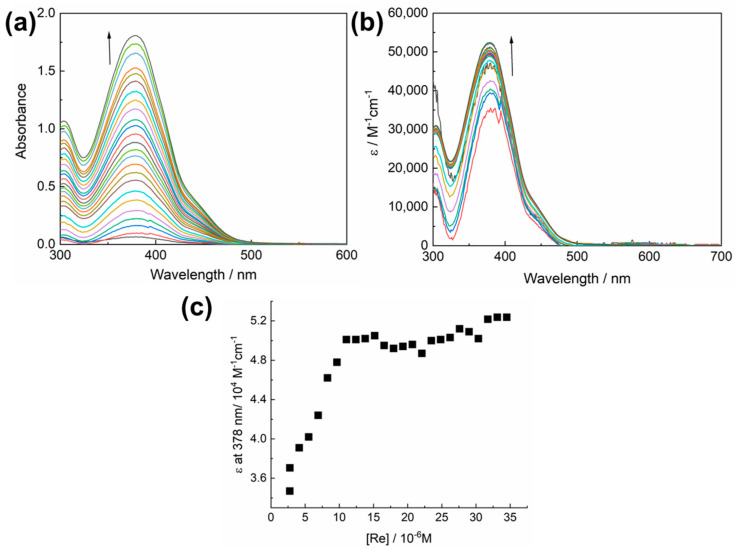
UV-vis absorption spectra displayed by plotting absorbance vs. wavelength (**a**) and apparent molar extinction coefficient ε vs. wavelength (**b**) for [Re(CO)_3_Cl(L)] DCM solutions with concentrations from 1.38 to 34.5 μM. Arrows indicate the spectral and ε changes upon increasing concentrations. (**c**) A plot of ε at 378 nm vs. the concentrations.

In order to further shed light on the aggregation behavior of [Re(CO)_3_Cl(L)], we explored the concentration-dependent UV–vis absorption and photoluminescence spectra. As shown in Figure 2, upon increasing the concentrations of [Re(CO)_3_Cl(L)] from 1.38 to 10 μM, a new shoulder at ~450 nm evolved, the values of full width at the half of the maximum became typically higher and the molar extinction coefficient ε increased with increasing concentrations up to a maximum ε of 5.24 M^−1^cm^−1^ at a concentration of ~10 μM (see Figure 2c). This ε value was little affected when concentrations further increased. The above observation revealed the successive formation of aggregates in the solutions with concentrations between 1.38 and 10 μM.

**Figure 3:**
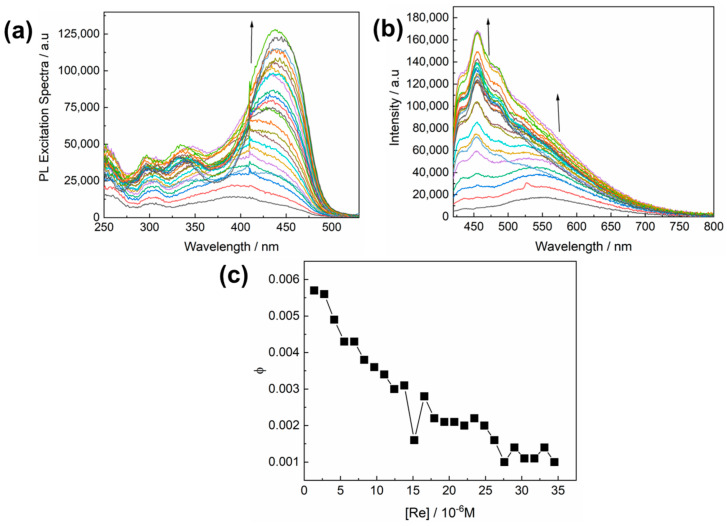
Concentration-dependent excitation spectra (λ_em_ = 500 nm) (**a**), emission spectra (λ_ex_ = 400 nm) (**b**) and a plot (**c**) of emission quantum yield vs. concentrations of [Re(CO)_3_Cl(L)] in DCM (1.38–34.5 μM). Note: arrows indicate the spectral changes upon increasing concentrations.

To confirm the results obtained by UV–vis absorption spectroscopy, concentration-dependent excitation and emission spectra of [Re(CO)_3_Cl(L)] in DCM were also measured. As clearly shown in Figure 3a, the excitation spectra show that the excitation intensities of [Re(CO)_3_Cl(L)] continually increased by increasing the solution concentrations. Moreover, significant red-shifts were observed for the excitation maxima in the low-energy region. As seen in Figure 3b, emission spectra also displayed significant changes with the concentration variations, namely, although the emission intensities were observed to increase with increasing concentrations, a new blue-shifted emission peak centered at 454 nm with substantially reduced emission quantum yields from 0.57% for 1.38 μM to 0.10% for 34.5 μM (see Figure 3c) evolved, revealing strong electronic coupling between [Re(CO)_3_Cl(L)] chromophores due to the aggregation formation. This conclusion is in close agreement with that from the absorption spectral analysis. The poorly emissive characteristic of the aggregation state of [Re(CO)_3_Cl(L)] could be a common aggregation behavior, which elicited a rapid non-radiative transition, thus leading to strong emission decay or quenching.

### 3.3. DFT Calculation

Enhancement in the separation of photo-generated electron–hole pairs has been reported to have a great effect on improving the photocatalytic performance of organic semiconductors [55,56,57]. To gain deep insights into the electronic structures and enhanced photoelectrocatalytic activities of [Re(CO)_3_Cl(L)], DFT calculations were carried out. The optimized structures (Figure S3) of [Re(CO)_3_Cl(phen)] (phen = 1,10-phenanthroline), the [Re(CO)_3_Cl(L)]-monomer and the [Re(CO)_3_Cl(L)]-dimer, the distributions of MOs (Figures S4 and S5) of the [Re(CO)_3_Cl(L)]-monomer and [Re(CO)_3_Cl(L)]-dimer and the partial molecular orbital energy levels (Figures S6 and S7, Table S1) of the Re(CO)_3_Cl(L)-monomer and [Re(CO)_3_Cl(L)]-dimer are presented in the Supporting Information. Computed data of the selected bond lengths and angles and dihedral angles for [Re(CO)_3_Cl(L)] are listed in Table S2, along with those abstracted from the crystal structure previously reported for an analogous Re complex [57]. As listed in Table S2, the computed geometric structures were proved to be rather reliable, as evidenced by the comparison of the optimized structural data of [Re(CO)_3_Cl(L)] with those of an analogous Re complex [57]. It is worth noting that [Re(CO)_3_Cl(L)] has a more pronounced delocalization of π-electron density than the parent complex of [Re(CO)_3_Cl(phen)]. As expected and previously reported [56,58], the introduction of the bithiophene group to [Re(CO)_3_Cl(phen)] can extend π-conjugation, which could promote the separation of photo-generated charges and enhance the light absorption into the visible spectrum, contributing to enhanced photoelectrocatalytic activity.

**Figure 4:**
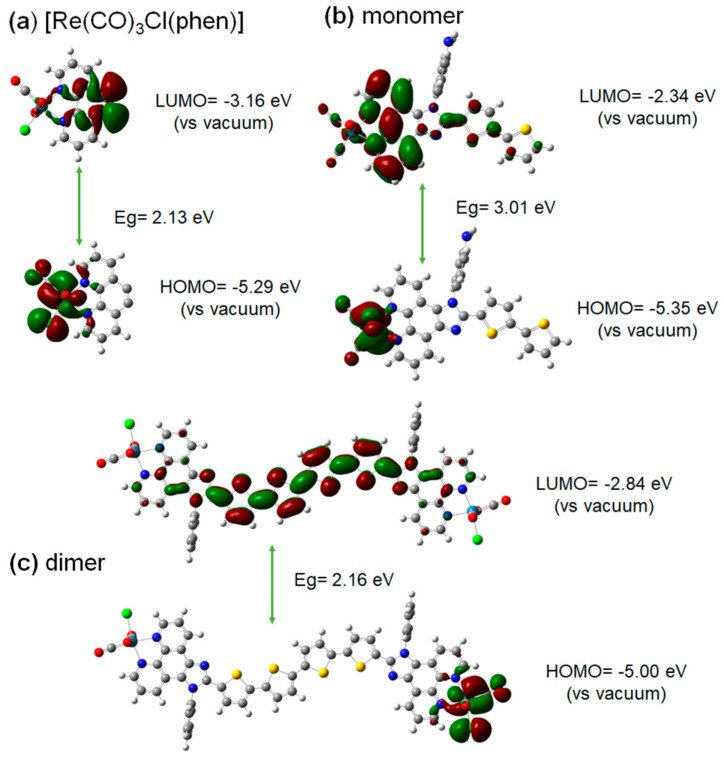
The distribution of frontier molecular orbitals of [Re(CO)_3_Cl(phen)] (**a**), [Re(CO)_3_Cl(L)]-monomer (**b**) and the [Re(CO)_3_Cl(L)]-dimer (**c**).

For the distribution of frontier molecular orbitals in the monomer and dimer of [Re(CO)_3_Cl(L)] (see Figures S4 and S5), the highest occupied molecular orbitals (HOMOs) are located over the d-orbital of the Re(I) center, proving the fact that the frontier orbital transitions occur mainly from the tricarbonyl rhenium(I) chloride moiety [59], but HOMO is only distributed on one rhenium center for the dimer. Moreover, the lowest unoccupied molecular orbital (LUMO) of the monomer is mainly distributed on the phenanthroline, and there is a partial distribution on the fused imidazole and thiophene rings. On the contrary, its thiophene rings markedly contribute to the LUMO of the dimer. As seen in Figure 4, Figures S6 and S7, the quantum mechanical calculations of the monomer and the dimer yielded HOMO levels of about −5.35 and −5.00 eV and LUMO levels of about −2.34 and −2.84 eV, resulting in HOMO–LUMO energy differences of about 3.01 and 2.16 eV, respectively. Obviously, the *E*_g_ of the dimer became narrower, which was beneficial for the light absorption of photocatalysts and the effective separation of the electron–hole pairs existing in the dimer, further improving photocatalytic/photoelectrocatalytic properties [57,58,60]. This speculation will be supported by the following PEC study.

### 3.4. Electrochemical Properties

**Figure 5:**
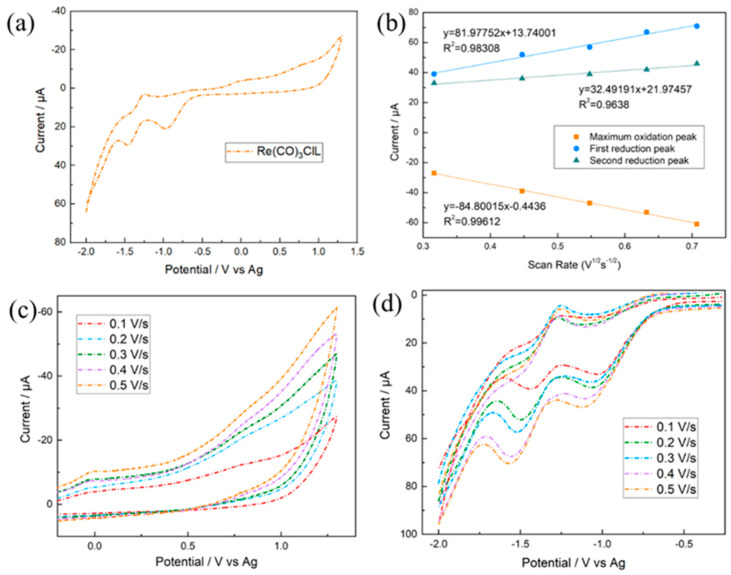
(**a**) CVs of 1 mM of [Re(CO)_3_Cl(L)] DCM solution under N_2_ at a scan rate of 0.1 V s^−1^. (**b**) Linear dependence of the cathodic peak current at the maximum oxidation peak or reduction peak vs. the square root of the scan rate. CVs of [Re(CO)_3_Cl(L)] in nitrogen-saturated DCM solution on positive (**c**) and negative (**d**) potential sweeps at potential scanning rates ranging from 0.1 V s^−1^ to 0.5 V s^−1^.

The electrochemical properties of [Re(CO)_3_Cl(L)] were studied by using cyclic voltammetry, and corresponding cyclic voltammograms (CVs) are shown in Figure 5a. Sweeping to negative potentials at 0.1 V s^−1^ yielded two reduction peaks with peak potentials at −0.95 and −1.46 V vs. Ag. The first reduction pointed convincingly to the oxidation of [Re(CO)_3_Cl(L)] aggregates, which caused the buildup of excess electrolytes in the polymer matrix and the subsequent discharge of these species [61,62]. The second reduction peak 2 was found to be at −1.46 vs. Ag, which is quasi-reversible and ascribed to be a ligand-based reduction with the added electron residing on L, furnishing [Re(CO)_3_Cl(L)**^.^**]^−^, and it may undergo dimerization by building an Re^0^-Re^0^ bond [63,64], as illustrated in following Equation (2):

Re^I^(CO)_3_ClL + e^−^ → [Re^I^(CO)_3_Cl(L)•]^−^ ↔ 1/2[(CO_3_)Cl(L)Re^0^-Re^0^L(Cl)(CO)_3_] + Cl^−^ (2)

When the potentials were scanned to positive potentials, the complex exhibited two ill-defined and irreversible oxidation peaks at +0.2 and +0.83 V, respectively. According to a previous electrochemical study, the first oxidation peak may originate from metal-centered Re(I/II) oxidations [65,66,67], while the second oxidation process was believed to involve the thiophene moieties, inducing their polymerization on the electrode surface [16,68]. Figure 5c,d present potential scan rate-dependent CVs of [Re(CO)_3_Cl(L)] in nitrogen-saturated DCM solution on positive sweep and negative potential sweep, respectively, with scanning rates ranging from 0.1 V s^−1^ to 0.5 V s^−1^. In all cases, the mentioned maximum oxidation peak current and reduction peak current stayed fully irreversible or reversible with no significant changes when the scan rates were altered. More importantly, the maximum oxidation and reduction peak currents were directly proportional to the square root of the scan rates with *R*^2^ > 0.96 (Figure 5b). This result revealed that a freely diffusing species in the solution obeyed the Randles–Sevcik Equation (3) [3,9,17].

(3) $$i_{max}=(2.69\times 10^{5})n^{\frac{3}{2}}D^{\frac{1}{2}}v^{\frac{1}{2}}C$$

Here, *i*_max_ is the maximum oxidation peak current or reduction peak current, *n* is the number of electrons involved in the redox reaction, *D* represents the diffusion coefficient of the complex, *v* is the critical scan rates (V s^−1^) and *C* stands for the concentration of the involved redox species.

### 3.5. PEC Properties

**Figure 6:**
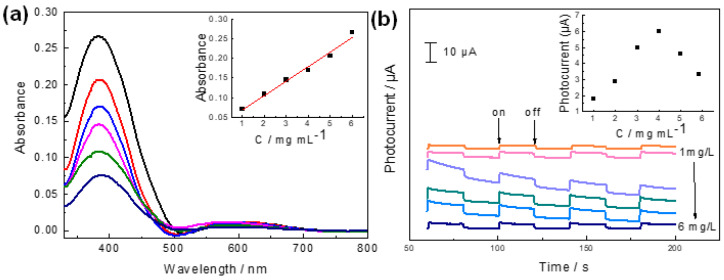
(**a**) The absorption spectra of the films prepared from [Re(CO)_3_Cl(L)] DCM solutions of varying concentrations of 1, 2, 3, 4, 5 and 6 mg mL^−1^ (from bottom to top). Inset: A plot of the absorbance of the films at 387 nm vs. concentrations of the Re complex in the drop-casting solutions. (**b**) Photocurrent responses of the films biased at −0.4 V vs. SCE under 100 mW/cm^2^ white light irradiation. Inset: A plot of the photocurrent vs. concentrations of the drop-casting solutions.

The photoelectrocatalysis of thin films is one of the most promising techniques for converting solar energy into electrical energy and chemical energy [69,70,71]. It is necessary to take into account a perfect balance between light absorption and charge transport from the active site to the supporting electrode for highly efficient PEC activity. Therefore, the thickness of the film is particularly important for the photoelectrocatalytic performance [15,72]. Thus, we prepared ITO electrodes drop-casted with [Re(CO)_3_Cl(L)] of varying thicknesses. Six different thicknesses of films were prepared by varying the DCM solution concentrations of [Re(CO)_3_Cl(L)] from 1 to 6 mg mL^−1^ (see Figure 6a). As shown in Figure 6b, all the films with varying thicknesses showed prompt and significant photocurrent responses to the on–off light illumination, implying the good PEC activity of the films. Additionally, the photocurrent saturation point was found to be reached for the film prepared from 4 mg/mL solution. On the contrary, when the casting solution concentrations were greater than or less than 4 mg/mL, the generated photocurrent decreased significantly. Therefore, the film prepared from a [Re(CO)_3_Cl(L)] concentration of 4 mg/mL was used for the following PEC studies.

**Figure 7:**
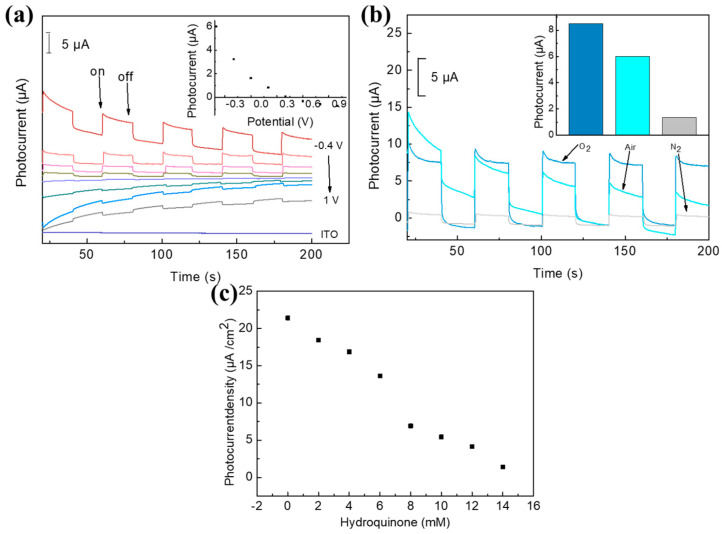
(**a**) Photocurrents responding to light irradiation on and off for bare ITO and the [Re(CO)_3_Cl(L)] film on ITO at bias potentials from top to bottom: −0.4, −0.2, 0, +0.2, +0.4, +0.6, +0.8 and +1 V vs. SCE. The inset: A plot of the photocurrent vs. the bias potentials. (**b**) Photocurrent responses and bar charts (inset) for the film in O_2_-, air- and N_2_-equilibrated electrolytes at a bias potential of –0.4 V vs. SCE. (**c**) Effects of the concentrations of the electron donor of hydroquinone on the photocurrent density of the film in air-equilibrated 0.1 M Na_2_SO_4_ at a bias potential of −0.4 V vs. SCE.

To further study the photocurrent polarity of the [Re(CO)_3_Cl(L)] film, we measured the photocurrent responses of the film with varying applied bias potentials from −0.4 to +1.0 V vs. SCE in N_2_ degassed 0.1 M Na_2_SO_4_ aqueous solution. As displayed in Figure 7a, photocurrents decreased evidently when the applied bias increased from −0.4 to +0.4 V vs. SCE, demonstrating that the film generated the cathodic photocurrents, and [Re(CO)_3_Cl(L)] acted as a typical p-type semiconductor. When the bias potentials further increased, photocurrents switched to weakly anodic, indicating that the film was of weak rectifying behavior. Moreover, when the photocurrent density of the film attained 0 μA/cm^2^, a bias potential of 0.2 V needed to be applied, implying that the open-circuit photovoltage of the film was +0.2 V. A maximum photocurrent (photocurrent density) of 6.0 μA (21.4 μA/cm^2^) was observed for the film biased at −0.4 V vs. SCE and immersed in air-equilibrated 0.1 M Na_2_SO_4_ solution, which outperformed what we previously reported for an Re(I) complex-based drop-casted film [15]. As shown in Figure 7b, the photocurrent (photocurrent density) was raised to 8.5 μA (30.4 μA/cm^2^) for [Re(CO)_3_Cl(L)]-coated ITO electrode biased at −0.4 V vs. SCE in oxygen saturated electrolyte solution, which is 6.1 times the photocurrent (photocurrent density) of 1.4 μA (5.0 μA/cm^2^) observed for that in the nitrogen-saturated electrolyte solution. These results proved that the photocurrents observed were cathodic. It is worth noting that the photocurrent (photocurrent density) observed at zero bias potential was found to be 1.6 μA (5.7 μA/cm^2^), which compares favorably with those for most of the previously reported thin films [73,74,75,76,77,78,79,80,81,82,83,84,85,86,87,88,89,90]. Thus, we conclude that the film we studied here may be a highly promising candidate for applications in PEC oxygen reduction or sensing.

**Figure 8:**
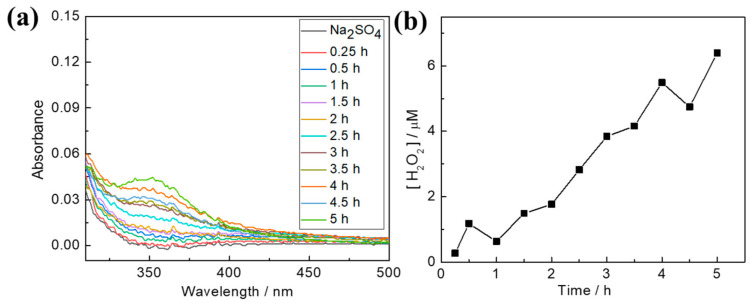
(**a**) UV–vis absorption spectra of molybdenum-triiodide indicator solution containing PEC cell solutions vs. PEC duration. (**b**) Production of H_2_O_2_ over time during photoelectrocatalysis in O_2_-equilibrated electrolytes at a bias potential of −0.4 V vs. SCE.

**Figure 9:**
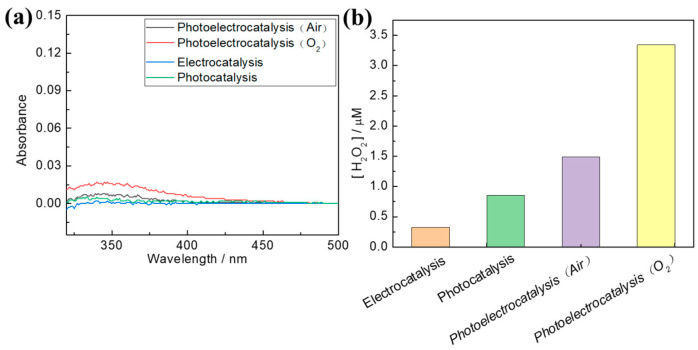
(**a**) Comparisons of UV–vis absorption spectra (**a**) and H_2_O_2_ concentration bar charts (**b**) of molybdenum-triiodide indicator solution containing 30-min electrocatalyzed, photocatalyzed and photoelectrocatalyzed solutions (air- and O_2_-equilibrated).

The above result opens up a possibility that the addition of electron donors or acceptors to the electrolyte solution could affect the directional movement of electrons in the modified film and electrolyte solution and further affect the generation of the photocurrent. In term of this, PEC experiments adding an electron donor of hydroquinone (H_2_Q) to the electrolyte solution were performed. As seen in Figure 7c, photocurrents were observed to decrease when the H_2_Q concentrations increased, further supporting the generation of the cathodic photocurrents. The result may be interpreted in light of the fact that the addition of the electron donor and acceptor to the electrolyte can inhibit or promote the directional electron movement from the ITO electrode to the electrolyte. Furthermore, we implemented a PEC oxygen reduction experiment with oxygen molecules in the electrolyte as electron acceptors. Photocathodes for the reduction of O_2_ to H_2_O_2_ have attracted increasing interest because of their potential advantages in terms of cost and safety [5,6,91]. Therefore, we examined whether the [Re(CO)_3_Cl(L)] film electrode could generate H_2_O_2_ in the above-mentioned PEC cell with an O_2_-equilibrated Na_2_SO_4_ aqueous solution under a bias potential of −0.4 V vs. SCE. The PEC production of H_2_O_2_ was confirmed using the spectrophotometric molybdenum-triiodide method [92] (see Figure 8). Figure 8a presents changes in the UV–vis absorption spectra of the molybdenum-iodide indicator solution, in which small aliquots of the PEC cell solutions were successively added. During 5 h photoelectrocatalysis, an absorption band with λ_max_ at 350 nm was detected, and the band intensities were found to increase with an increasing photoelectrocatalysis duration, indicating the formation of H_2_O_2_ in the PEC cell solution. The H_2_O_2_ concentrations were quantified according to the working curves, as shown in the Figure S8 insets. As shown in Figure 8b, a maximum H_2_O_2_ concentration of 6.39 μM was obtained after 5 h photoelectrocatalysis. Notably, the H_2_O_2_ concentration almost linearly increased over 5 h, implying that the film was robust against peeling off the electrode surface for at least 5 h. It is also important to note from Figure S9 that the UV–vis absorption spectra for [Re(CO)_3_Cl(L)] based ITO electrode before and after 5 h photoelectrocatalysis did not show obvious changes, indicating that [Re(CO)_3_Cl(L)] was of good PEC stability. The H_2_O_2_ production by photoelectrocatalysis, electrocatalysis and photocatalysis was also comparatively studied under similar experimental conditions. As shown in Figure 9, all three methods showed a positive hydrogen peroxide production, but the PEC production of H_2_O_2_ was much more efficient than the addition of the photocatalysis and the electrocatalysis, indicating that photo- and electro-catalysis synergistically enhanced the production of H_2_O_2_. The above results indicate that [Re(CO)_3_Cl(L)]-based film electrode has the advantages of simple production, high stability and a low cost and serves as an interesting parent complex for developing more efficient photoelectrocatalysts for O_2_ reduction or sensing or photoelectrocatalytic electrodes for H_2_O_2_ production. 

**Figure 10:**
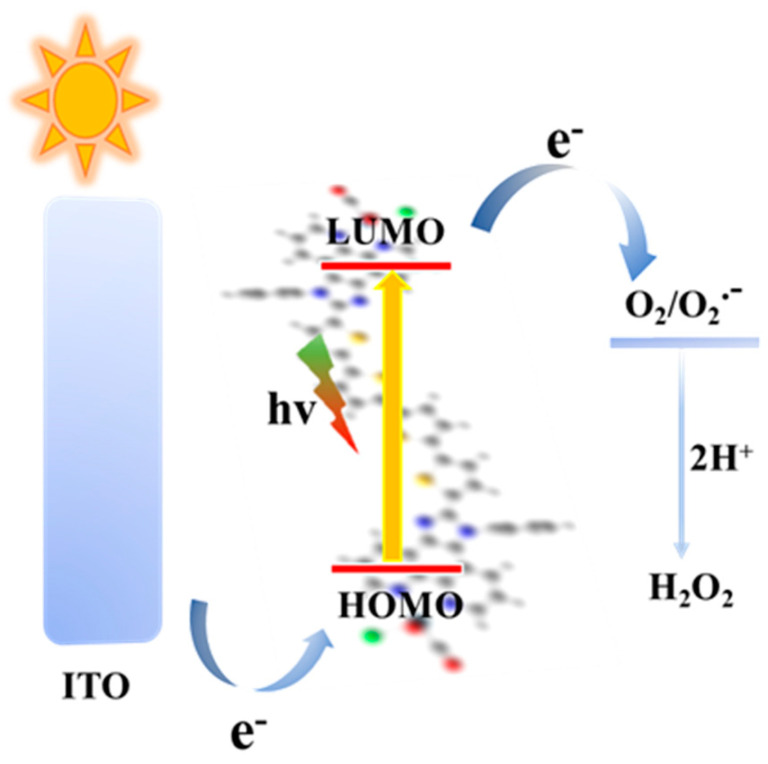
Proposed electron transfer mechanism of [Re(CO)_3_Cl(L)] on ITO for cathodic photocurrent generation and photoelectrocatalytic H_2_O_2_ production.

Based on previous reports [1,93] and our current results [23,94], the mechanism of cathodic photocurrent generation and H_2_O_2_ synthesis is postulated as follows (Figure 10): light irradiation of the rhenium complex excited the electron from HOMO into LUMO to form the excited state complex, which further transferred the excited electrons to electron acceptors (O_2_) in the electrolyte solution; the oxidized [Re(CO)_3_Cl(L)] was regenerated by accepting the electron from the conduction band of ITO. In this case, the donor present in the electrolyte would be certainly unfavorable for the cathode photocurrent generation. The combination of photoelectrochemically generated O_2_⦁^−^ with protons in the electrolyte solution yielded H_2_O_2_ (see Equations (4) and (5)).

O_2_ + 2[Re(CO)_3_Cl(L)]* → O_2_•^−^ + 2[Re(CO)_3_Cl(L)]*•^+^ (4)

O_2_•^−^ + 2H^+^ → H_2_O_2_ (5)

## 4. Conclusions

In summary, a novel Re(I)-phenanthroline complex with a bithiophene group, [Re(CO)_3_Cl(L)], is synthesized and demonstrated to exhibit obvious aggregation behavior in DCM solutions at high concentrations and in its drop-casted films. The experimental results and DFT calculations indicated that introduction of the bithiophene group resulted in enhanced conjugation. [Re(CO)_3_Cl(L)] drop-casted film modified ITO electrode-based PEC cells at a bias potential of −0.4 V vs. SCE containing N_2_- and O_2_-saturated electrolyte solution exhibited a maximum cathodic photocurrent density of 5.0 and 30.4 μA/cm^2^, respectively, demonstrating that the PECs exhibited an intriguing photoelectrocatalytic O_2_ reduction property. More importantly, the photoelectrocatalytic O_2_ reduction product was evidenced to be H_2_O_2_, with a maximum [H_2_O_2_] of 6.39 μM achieved after 5 h of photoelectrocatalysis. The results would guide the design of more efficient Re complex-based film electrodes for applications in PEC O_2_ sensing and H_2_O_2_ production, related energy storage and conversion and electrochemiluminescence [95].

## Data Availability

The data presented in this study are available on request from the corresponding author.

## Supplementary Materials

The following supporting information can be downloaded at: https://www.mdpi.com/article/10.3390/molecules28073229/s1. Figure S1: Comparison of ^1^H NMR spectra of ligand L (top panel) and the complex Re(CO)_3_ClL (bottom panel) in (CD_3_)_2_SO (proton resonance peaks of thiophene, benzene and o-phenanthroline rings are labeled as ,  and , respectively); Figure S2: IR spectra of L (top line) and Re(CO)_3_ClL (bottom line) in KBr pellets; Figure S3: Optimized structures of Re(CO_3_)(o-Phen)Cl (a), Re(CO)_3_ClL-monomer (b) and Re(CO)_3_ClL- dimer (c) from DFT calculations. Cl green, Re cyan, O red, C gray, N blue, S yellow, H white; Figure S4: The distribution of MOs of Re(CO)_3_ClL- monomer. Cl green, Re cyan, O red, C gray, N blue, S yellow, H white; Figure S5: The distribution of MOs of Re(CO)_3_ClL- dimer. Cl green, Re cyan, O red, C gray, N blue, S yellow, H white; Figure S6: Partial molecular orbital energy levels of Re(CO)_3_ClL-monomer calculated by DFT; Figure S7: Partial molecular orbital energy levels of Re(CO)_3_ClL-dimer calculated by DFT; Figure S8: The UV-Vis absorption spectra of H_2_O_2_ at different concentrations, the top inset shows the dependence of absorbance at 350 nm on H_2_O_2_. (a) [H_2_O_2_] is 1–10 × 10^−6^ M, (b) [H_2_O_2_] is 1–10 × 10^−5^ M; Figure S9: Normalized UV-Vis absorption spectra of a CH_2_Cl_2_ solution (blue line), and drop-coated film of Re(CO)_3_ClL before (black line) and after (red line) photoelectrocatalysis for 5 h; Table S1: Computational energy levels of Re(CO)_3_ClL-monomer; Table S2: Comparison of computational selected bond lengths (Å), bond angles (°), and dihedral angels (°) of Re(CO)_3_ClLwith the atomic labelling scheme (left) and the molecular structure (right) shown below this table using the DFT-B3LYP at the LanL2DZ level with those of crystal structure of fac-[ReBr(CO)_3_(L_3_)], the Ref crystal, which was reported in text Ref. [57].

## Sample Availability

Samples of the compounds [Re(CO)_3_Cl(L)] and [Re(CO)_3_Cl(phen)] are available from the authors.
