# Supplementary material for: Photoelectrocatalytic Dioxygen Reduction Based on a Novel Thiophene-Functionalized Tricarbonylchloro(1,10-phenanthroline)rhenium(I)
Source: Molecules. 2023 Apr 4;28(7):3229. doi: 10.3390/molecules28073229 (PMC10096527; doi:10.3390/molecules28073229)
Supplement: Supplementary file 1 [file molecules-28-03229-s001.zip › molecules-2251940-supplementary.pdf]

# Supplementary Material

# Photoelectrocatalytic Dioxygen Reduction based on a Novel Thiophene-Functionalized Tricarbonylchloro(1,10-phenanthroline)rhenium(I)

Yu-Qin Li and Ke-Zhi Wang,*

Beijing Key Laboratory of Energy Conversion and Storage Materials, College of Chemistry, Beijing Normal University, Beijing 100875, China

*kzwang@bnu.edu.cn; Tel.: +86-10-58805476; Fax: +86-10-58802075

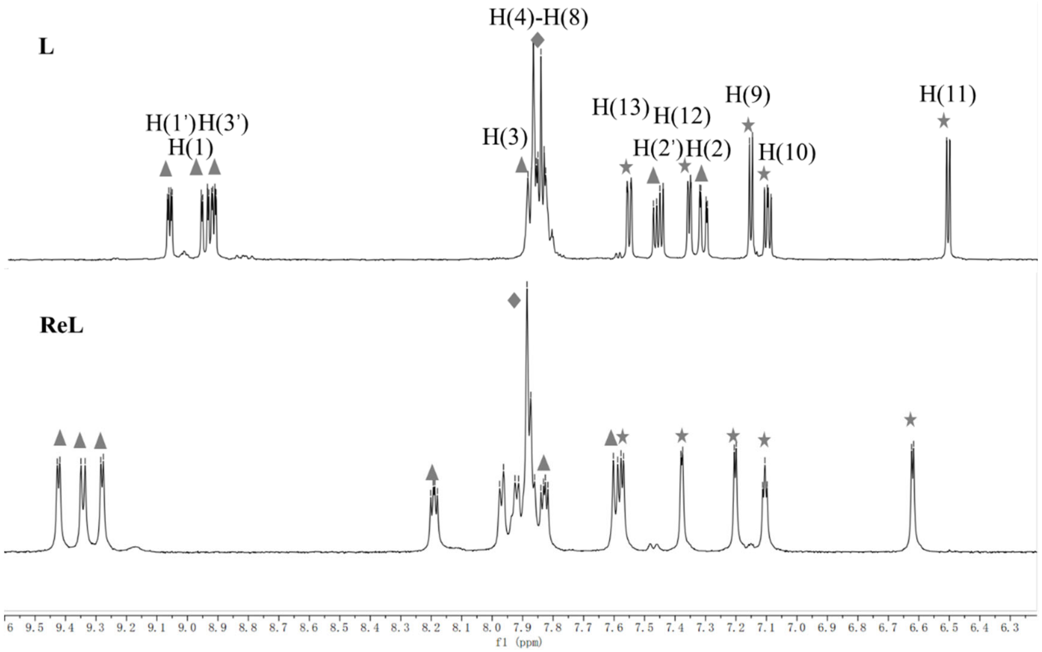


**Figure S1.** Comparison of $^{1}H$ NMR spectra of ligand L (top panel) and the complex $[Re(CO)_3Cl(L)]$ (bottom panel) in $(CD_3)_2SO$ (proton resonance peaks of thiophene, benzene and 1,10-phenanthroline rings are labeled as ★, ◆ and ▲, respectively). Atom numbering schemes are shown below:

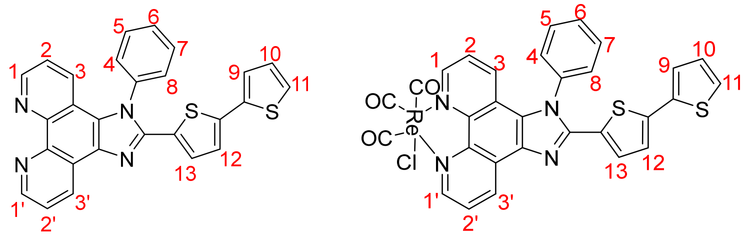

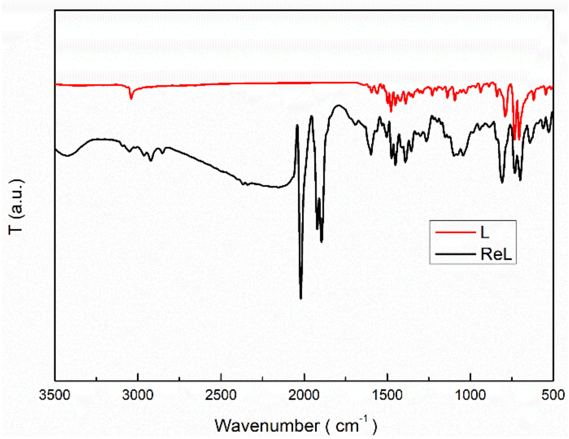


**Figure S2.** IR spectra of L (top line) and [Re(CO)$_3$Cl(L)] (bottom line) in KBr pellets.

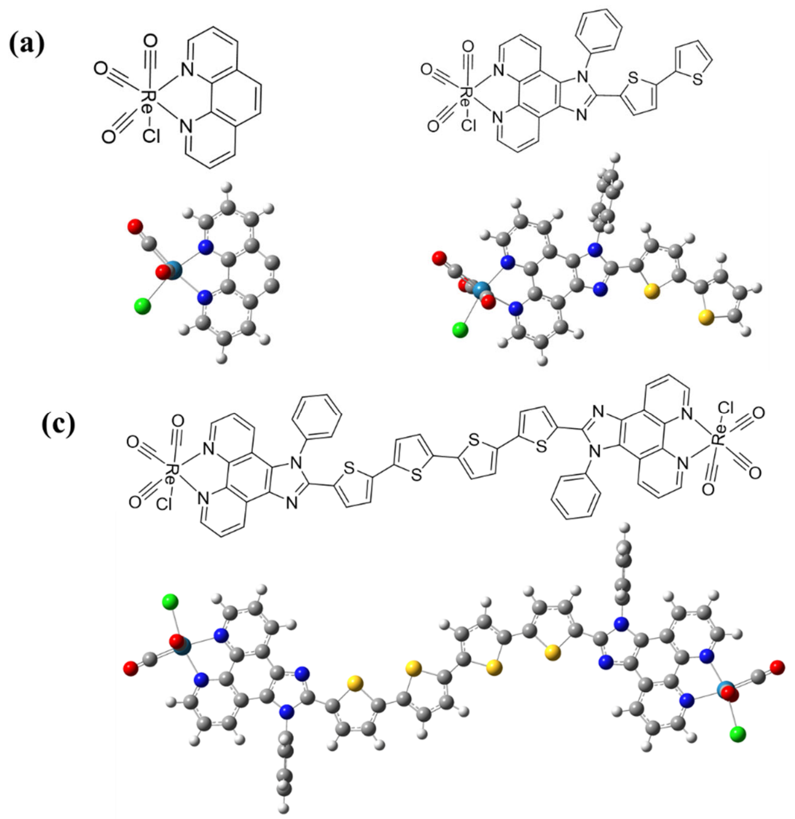


**Figure S3.** Optimized structures of [Re(CO)$_3$Cl(phen)] (a), [Re(CO)$_3$Cl(L)]-monomer (b) and [Re(CO)$_3$Cl(L)]-dimer (c) from DFT calculations. Cl green, Re cyan, O red, C gray, N blue, S yellow, H white.

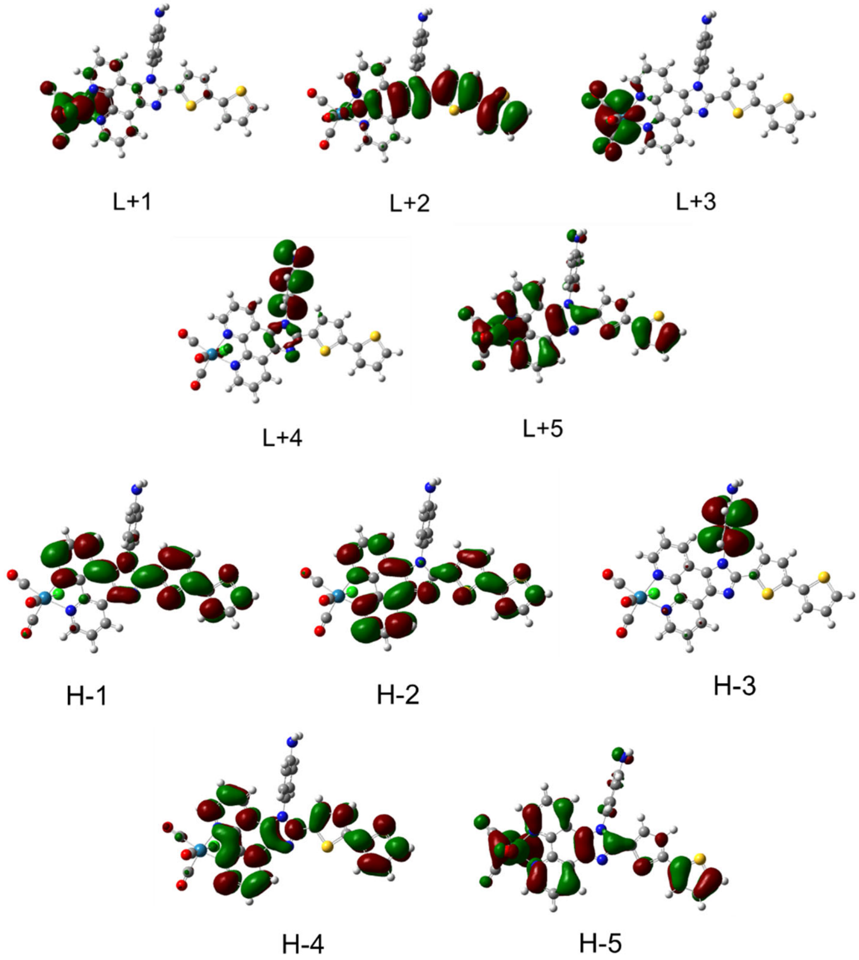


**Figure S4.** The distribution of MOs of [Re(CO)$_3$Cl(L)]-monomer. Cl green, Re cyan, O red, C gray, N blue, S yellow, H white.

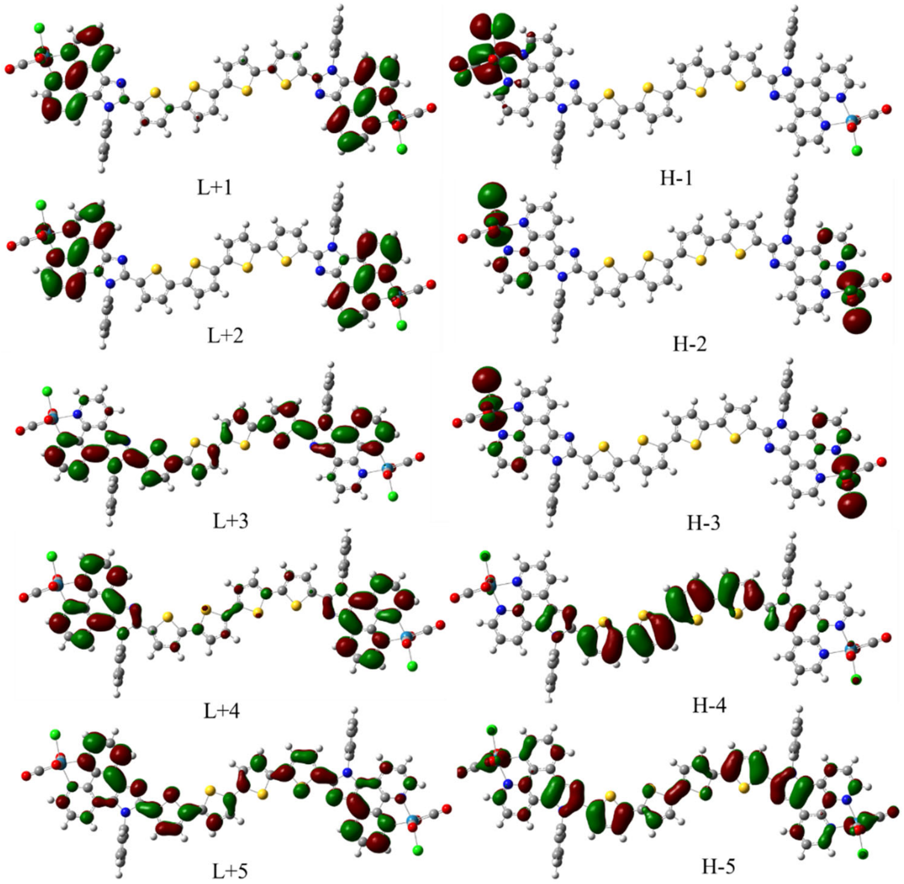


**Figure S5.** The distribution of MOs of [Re(CO)$_3$Cl(L)]-dimer. Cl green, Re cyan, O red, C gray, N blue, S yellow, H white.

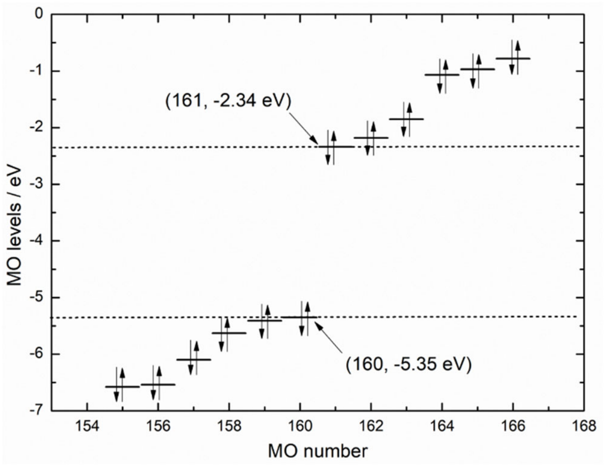


**Figure S6.** Partial molecular orbital energy levels of [Re(CO)$_3$Cl(L)]-monomer calculated by DFT.

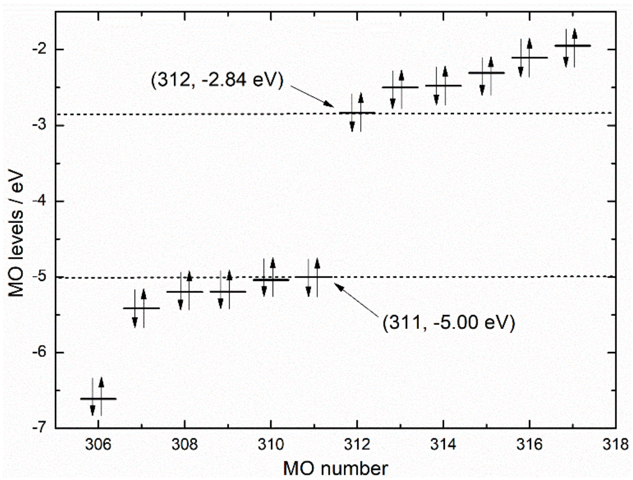


**Figure S7.** Partial molecular orbital energy levels of $[Re(CO)_3Cl(L)]$-dimer calculated by DFT.

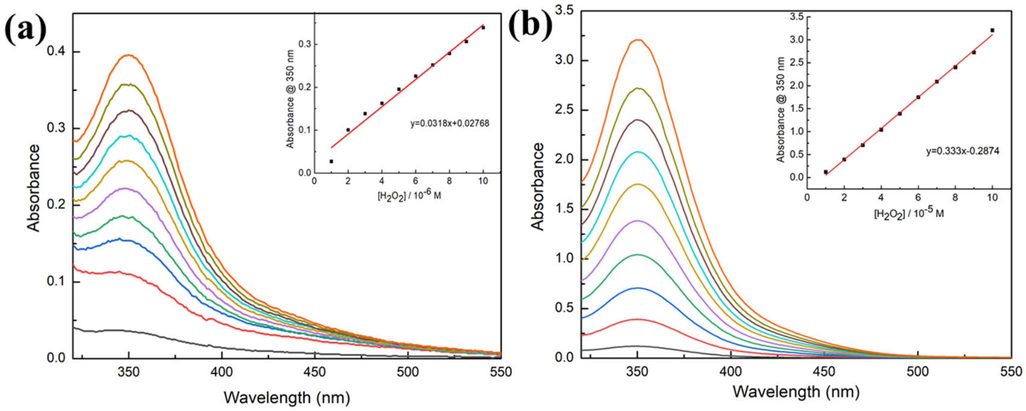


**Figure S8.** UV-vis absorption spectra of molybdenum-triiodide indicator solution containing different concentrations of $H_2O_2$, the top inset shows the dependence of absorbance at 350 nm on $H_2O_2$. (a) [$H_2O_2$] is 1-10×$10^{-6}$ M, (b) [$H_2O_2$] is 1-10×$10^{-5}$ M.

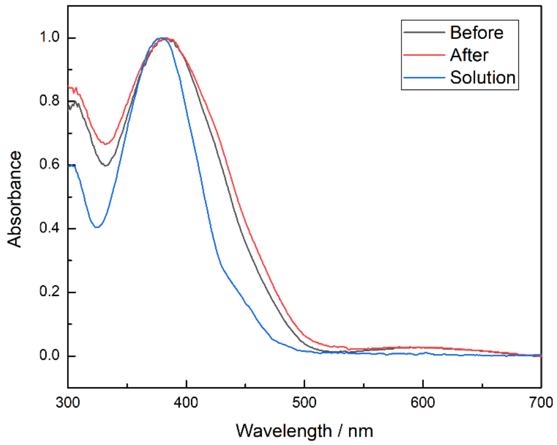


**Figure S9.** Normalized UV-vis absorption spectra of a $CH_2Cl_2$ solution (blue line), and drop-coated film of [Re(CO)$_3$Cl(L)] before (black line) and after (red line) photoelectrocatalysis for 5 h.

**Table S1** Computational energy levels of $[Re(CO)_3Cl(L)]$-monomer.

| H-2 | H-1 | HOMO | LUMO | L+1 | L+2 | $\Delta_{L-H}$ | $\Delta_{L-H-1}$ | $\Delta_{L-H-2}$ | Eg |
|---|---|---|---|---|---|---|---|---|---|
| -5.63 | -5.41 | -5.35 | -2.34 | -2.18 | -1.85 | 3.01 | 3.05 | 3.27 | 2.54 |

HOMO (H) : Energy levels of highest occupied molecular orbital.
LUMO (L) : Energy levels the lowest unoccupied molecular orbital.
H-1(2) : the second (third) HOMO level.
L+1(2) : the second (third) LUMO level.
$\Delta_{L-H}$ : $E_{LUMO}$-$E_{HOMO}$.
Eg : the optical gaps of the complex from UV-vis absorption spectra.

First, the coordination bond length of o-phenanthroline is slightly longer than that of carbonyl ligand and shorter than that of chlorine ligand, which is similar to crystal coordination bond length of fac-$[ReBr(CO)_3(L_3)]$ [S1]. Second, imidazole is almost coplanar with thiophene ring and has a torsion angle of -0.614°, whilst phenylamine group is almost orthogonal to imidazole ring with a torsion angle of -89.504. By comparing the energy of the UV-vis absorption spectra with theory calculation, we can find that DFT calculation gave higher energy difference than experimentally determined, which is attributed to the fact that the results obtained by calculation are relative to the vacuum system, whereas the experimental results were obtained in the relatively complex system, namely experimental system possesses molecular solvation, geometrical changes and polarization in aqueous solution and so on.

**Table S2** Comparison of computational selected bond lengths (Å), bond angles (°), and dihedral angels (°) of $[Re(CO)_3Cl(L)]$ with the atomic labelling scheme (left) and the molecular structure (right) shown below this table, with those of crystal structure of fac-$[ReBr(CO)_3(L_3)]$, a previously reported Ref crystal [S1].

| Complex | Bond lengths | | | | | | Bond angles | | | [f]Dihedral angles | |
|---|---|---|---|---|---|---|---|---|---|---|---|
| | [a]Re-$CO^1$ | [a]Re-$CO^2$ | [a]Re-$CO^3$ | [b]Re-Cl/Br | [c]Re-$N^1$ | [c]Re-$N^{2(1)}$ | [d]$A_N$ | [e]$Aco_{13}$ | [e]$Aco_{12}$ | $N_5$-$C_6$-$C_7$-S8 | $C_6$-$N_5$-$C_{13}$-$C_{14}$ |
| $[Re(CO)_3Cl(L)]$ (calc.) | 1.927 | 1.927 | 1.919 | 2.521 | 2.211 | 2.205 | 74.7 | 91.9 | 90.4 | -0.61 | -89.5 |
| Ref crystal | 1.913 | 1.935 | 1.926 | 2.552 | 2.169 | 2.176 | 75.3 | 92.2 | 87.2 | - | - |

[a]Re-$CO^n$( $n$ = 1-3) expresses the coordination bond length between Re and carbonyl group.

[b]Re-Cl/Br expresses the coordination bond length between Re and Br or Cl.

[c]Re-$N_{1(2)}$ expresses the coordination bond length between Re and N atoms of phenanthroline.

[d]$A_N$ expresses the coordination bond angle between central Re and two N atoms of the phenanthroline.

[e]Aco expresses the coordination bond angle between central Re and C atoms of carbonyl group.

[f]The dihedral angles expresses an angle formed by two planes composed of the corresponding four atoms.

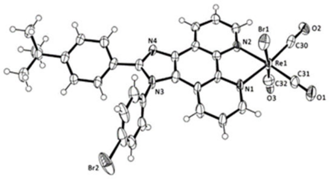

**Reference**

S1. Bonello, R.O.; Morgan, I.R.; Yeo, B.R.; Jones, L.E.J.; Kariuki, B.M.; Fallis, I.A.; Pope, S.J.A. Luminescent rhenium(I) complexes of substituted imidazole[4,5-f]-1,10-phenanthroline derivatives. *J. Organomet. Chem.* **2014**, *749*, 150–156.
